# Natural Product Quercetin-3-methyl ether Promotes Colorectal Cancer Cell Apoptosis by Downregulating Intracellular Polyamine Signaling

**DOI:** 10.7150/ijms.93903

**Published:** 2024-03-25

**Authors:** Jincheng Zeng, Yuancheng Zhang, Yuming Fang, Jiachun Lian, Hailiang Zhang, Shaobing Zhang, Bihua Lin, Ziyu Ye, Caihong Li, Xianxiu Qiu, Yanfang Liang

**Affiliations:** 1 Dongguan Key Laboratory of Medical Bioactive Molecular Developmental and Translational Research, Guangdong Provincial Key Laboratory of Medical Molecular Diagnostics, Guangdong Medical University, Dongguan 523808, China.; 2 Department of Pathology, Binhaiwan Central Hospital of Dongguan, Dongguan 523000, China.; 3 Dongguan Proof-of-Concept Centers for Medical Use, Guangdong Xinghai Institute of Cell, Dongguan 523808, China.; 4 Department of Clinical Laboratory, Yuedong Hospital, The Third Affiliated Hospital of Sun Yat-sen University, China.

**Keywords:** natural product, quercetin-3-methyl ether, colorectal cancer cell, cell apoptosis

## Abstract

Dysregulation of cellular metabolism is a key marker of cancer, and it is suggested that metabolism should be considered as a targeted weakness of colorectal cancer. Increased polyamine metabolism is a common metabolic change in tumors. Thus, targeting polyamine metabolism for anticancer therapy, particularly polyamine blockade therapy, has gradually become a hot topic. Quercetin-3-methyl ether is a natural compound existed in various plants with diverse biological activities like antioxidant and antiaging. Here, we reported that Quercetin-3-methyl ether inhibits colorectal cancer cell viability, and promotes apoptosis in a dose-dependent and time-dependent manner. Intriguingly, the polyamine levels, including spermidine and spermine, in colorectal cancer cells were reduced upon treatment of Quercetin-3-methyl ether. This is likely resulted from the downregulation of SMOX, a key enzyme in polyamine metabolism that catalyzes the oxidation of spermine to spermidine. These findings suggest Quercetin-3-methyl ether decreases cellular polyamine level by suppressing SMOX expression, thereby inducing colorectal cancer cell apoptosis. Our results also reveal a correlation between the anti-tumor activity of Quercetin-3-methyl ether and the polyamine metabolism modulation, which may provide new insights into a better understanding of the pharmacological activity of Quercetin-3-methyl ether and how it reprograms cellular polyamine metabolism.

## 1. Introduction

Colorectal cancer (CRC) is the third most common cancer in the world, and its morbidity and mortality are increasing globally 1. CRC accounted for 10% of cancer cases and 9.4% of cancer deaths worldwide in 2020, and the number of new CRC cases worldwide is expected to reach 3.2 million in 2040^2^. Colorectal cancer is a multifactorial disease involving genetic, epigenetic, and environmental factors 3. The information provided by genetic and transcriptomic studies has greatly facilitated the diagnosis and treatment of colorectal cancer 4, 5. However, advanced treatment of colorectal cancer becomes challenging and resistance to most forms of combination therapy develops, leading to CRC metastasis as the leading cause of cancer-related death 6, 7. Therefore, in order to better propose targetable vulnerabilities for colorectal cancer, it is necessary to understand the mechanisms that lead to colorectal cancer initiation, progression, and dissemination.

Cell metabolism is closely linked to tumor cell fate and phenotype. Work done over the past 17 years has demonstrated that the reprogramming of cellular metabolism is the active process controlled by oncogenes and tumor suppressors that provide cancer cells with energy, reduction of equivalents and biosynthetic precursors 8. In fact, cancer cells can maintain their high cell division rate and biosynthetic demands by utilizing most of the core metabolic pathways, including glucose, amino acid and lipid metabolism 9. Polyamine metabolism, which is often dysregulated in cancer and other hyperproliferative diseases, has become an attractive target for cancer therapeutic interventions 10. Polyamines are a class of aliphatic polycationic compounds found in mammals, consisting mainly of putrescine (PUT), spermidine (SPD) and spermine (SPM) 11. It has been shown that polyamines have important roles in regulating apoptosis, cell division and differentiation, cell viability, DNA and protein synthesis, gene expression, homeostasis and signal transduction *in vivo* 12-14. Polyamine biosynthesis begins with arginine. In mammals, arginine is first converted to ornithine by arginase, then decarboxylated by ornithine decarboxylase (ODC) to synthesize putrescine, which then synthesizes spermidine and spermine in turn under the action of spermidine synthase (SRM) and spermine synthase (SMS) 11, 12, 15. To date, numerous studies have shown that polyamine metabolism disorders are closely related to many diseases, including cancer, inflammation, atherosclerosis, renal failure and diabetes. Excessive accumulation or depletion of intracellular polyamines, especially SPD, can have deleterious effects on mammalian cells and may lead to cell death 10, 12, 16-19. A number of inhibitors targeting polyamine biosynthetic and catabolic enzymes and polyamine transport have been developed and tested in preclinical studies 20. Despite early *in vitro* trials and several preclinical cancer models showing promise, clinical trials using a single drug to target the polyamine pathway have not worked well. Fortunately, 2-difluoromethylornithine (DFMO), an irreversible ODC inhibitor, has shown single significant success in the treatment of anaplastic gliomas 20, 21, and DFMO in combination with sulindac in the prevention of colorectal cancer (CRC) has shown encouraging efficacy in clinical trials 22. Using this negative regulation of polyamines content in cancer cells, analogues similar to natural polyamines have been developed, which are sufficient to simulate this effect and reduce the content in cancer cells, but cannot play their tumor-promoting function. A highly innovative application of polyamine analogs is for the construction of self-immolative prodrug nanoparticles for delivery of therapeutic nucleic acids and other drug cargoes 23, 24. Particularly exciting is that polyamine blockade therapy (PBT) promotes antitumor immune responses, resulting in greater antitumor effects than would be expected from depleting polyamines alone in tumor cells 25. Over the past decade, the emergence of new research tools and methods has allowed us to better understand the molecular mechanisms involved in polyamines and how to best target polyamine metabolism and function for therapeutic benefit in cancer treatment.

In recent decades, many countries have utilized multiple forms of complementary and alternative medicine to combat cancer 26, 27. Quercetin-3-methyl ether (Q3ME), a natural compound found in various plants, including *Caesalpinia Bonduc, Larrea Divaricata Cav., Cistus Laurifolius, Polygonum amphibium* 28-31, has been shown to have anti-oxidant, anti-inflammatory, anti-viral, hypoglycemic and immunomodulatory effects 32-36. Most interestingly, Quercetin-3-methyl ether has obtained wide attention in cancer treatment. Studies have shown that it significantly inhibits the formation of human breast cancer stem cells by inhibiting Notch1 and Pl3K/Akt signaling pathways, and Q3ME can also induce lymphoma cell apoptosis through nitrosative stress 37, 38. However, the effect of Quercetin-3-methyl ether on polyamine metabolism in colorectal cancer cell is largely unknown. In the present study, we demonstrated the tumor-suppressing effect of Quercetin-3-methyl ether by inhibiting colorectal cancer cell viability, and inducing apoptosis. We also revealed that the apoptosis-inducing activity of Q3ME is correlated with polyamine metabolism modulation, which reduces cellular polyamine levels by suppressing SMOX (spermine oxidase) expression.

## 2. Materials and Methods

### 2.1. Reagents and Antibodies

Quercetin-3-methyl ether was purchased from Sigma-Aldrich. Dulbecco's modified Eagle's medium (DMEM) was obtained from Gibco. Fetal bovine serum (FBS) was obtained from Biological Industries. Bcl-xL antibody (2764P), Bax antibody (5023S), Cleaved Caspase-3 antibody (9664S), Caspase-3 antibody (9662S), and GAPDH (14C10) were purchased from Cell Signaling Technology. SMOX antibody (15052-1-AP), ODC1 antibody (28728-1-AP), SRM antibody (15979-1-AP) were purchased from Proteintech.

### 2.2. Cell culture

Human colorectal cancer cell line RKO and SW1116 were obtained from the Cell Bank Type Culture Collection of the Chinese Academy of Sciences (Shanghai, China). RKO is a poorly differentiated colon carcinoma cell line and exhibited an epithelial-like morphology. SW1116, a cell line exhibiting epithelial morphology, was isolated from the colorectal adenocarcinoma. RKO and SW1116 cells were cultured in DMEM medium containing 10% fetal bovine serum and 1% penicillin/streptomycin in a 5% CO_2_ incubator at 37°C.

### 2.3. Cell growth assay

To examine the effect of Quercetin-3-methyl ether on colorectal cancer cell viability, the cells were seeded (3×10^3^ cells/well) in 96-well plates with DMEM containing 10% FBS, and cultured at 37°C in a 5% CO_2_ incubator. After 24 h, the cells were supplemented with fresh medium and treated with Quercetin-3-methyl ether (0 µM, 10 µM, 20 µM, 40 µM). Following culturing for the indicated times (0-72 h), 10 µL of CCK-8 solution (Dojindo) was added into each well and the wells were cultured at 37°C in a 5% CO_2_ incubator for 2 hours. Finally, the absorbance was measured at 450 nm.

### 2.4. Western blot analysis

The cancer cells (6×10^5^ cells/well) were cultured overnight in a 6-well plate, the cells were then treated with Quercetin-3-methyl ether (0 µM, 10 µM, 20 µM) in a medium containing 10% FBS. The collected cells were lysed on ice with lysis buffer and then ultrasonically lysed. Centrifugation was performed at 12000 rpm at 4°C for 15 min, and the supernatant was collected for further analysis. Total protein concentration of cell lysates was determined using the BCA Protein Concentration Assay Kit (Enhanced) (Beyotime Biotechnology) according to the manufacturer's instruction. Protein samples were separated by SDS-PAGE and transferred to PVDF membrane (Millipore), where they were incubated with primary and HRP-conjugated secondary antibodies. The protein bands were observed with ECL reagent. GAPDH was served as the loading control.

### 2.5. Apoptosis assay

RKO and SW1116 cells (5×10^5^ cells/well) were inoculated into a 6-well plate and incubated overnight in an incubator with 5% CO_2_ at 37°C. The cells were treated with Quercetin-3-methyl ether (0 µM, 10 µM, 20 µM). Apoptotic cells were analyzed using Annexin V FITC/PI staining following the manufacturer's protocol and the fluorescence intensity of the staining was measured by flow cytometry.

### 2.6. Colony formation assay

The cells were inoculated at a density of 500 cells per well in a 6-well plate and were allowed to incubate overnight in a 5% CO_2_ incubator at 37°C. Subsequently, the cells were provided fresh culture medium and exposed to Quercetin-3-methyl ether (0 µM, 10 µM, 20 µM). The medium was refreshed every 3 days. After a 7-day incubation period, the colonies were fixed using 10% formaldehyde for 10 min and stained with a 1% crystal violet solution for 25 min. Subsequently, images were captured and the colonies were quantified.

### 2.7. RNA extraction and RT-qPCR

Total RNA was extracted using RNA isotriazole reagent (TaKaRa, Japan). Reverse transcription of RNA was performed using the PrimeScript™ RT Reagent Kit and gDNA Eraser (TaKaRa, Japan) according to the manufacturer's instructions. Real-time PCR was generated on an Applied Biosystems 7500 (Thermo Fisher, USA) using Master Mix containing SYBR Green (TaKaRa, Japan). *GAPDH* was used as a control. Relative expressions were calculated using the comparative threshold cycle (2-ΔΔCt) method. The primers were as follows: *ODC1* forward primer: TTTACTGCCAAGGACATTCTGG, reverse primer: GGAGAGCTTTTAACCACCTCAG; *SRM* forward primer: ACCAGCTCATGAAGACAGCACTCA, reverse primer: TGCTACACAGCATGAAGCCGATCT; *SMOX* forward primer: TCTGCACAGAGATGCTTCGACAGT, reverse primer: TTGAGCCCACCTGTGTGTAGGAAT; *GAPDH* forward primer: GCACCGTCAAGGCTGAGAAC, reverse primer: TGGTGAAGACGCCAGTGGA.

### 2.8. Spermine and spermidine analysis by HPLC

The levels of spermine and spermidine in colorectal cancer cells were determined by high performance liquid chromatography (HPLC). RKO and SW1116 cells (5×10^5^ cells / well) were inoculated in a six-well plate and incubated with Quercetin-3-methyl ether (0 µM -20 µM). The culture medium treated with Quercetin-3-methyl ether was collected to measure extracellular spermine and spermidine levels. The cells in the 6-well plate were digested by trypsin and centrifuged. Cold 5% HClO_4_ was added to the cell samples to precipitate protein. After low-temperature centrifugation, spermine and spermidine levels in the cells were measured in the supernatant. Immediately after derivatization with Dansyl chloride, it was determined by HPLC system (Shimadzu, Japan). Chromatographic conditions: Mobile phase: A phase was ultrapure water, B phase was acetonitrile, gradient elution; Detector: fluorescence detector; excitation wavelength is 340 nm, emission wavelength is 510 nm; Flow rate: 1ml/min; injection volume: 10 μL; Column temperature: 40°C.

### 2.9. Statistical analysis

All numerical data are presented as mean ± standard error of the mean (SEM). Statistical significance of differences was performed by Student's* t*-test using GraphPad Prism 8.0 software (USA). One-way ANOVA was used to determine statistical differences between multiple tests. Values of *P*<0.05 were considered to be significantly different. All experiments were repeated three times.

## 3. Results

### 3.1. Quercetin-3-methyl ether inhibits the viability of colorectal cancer cells

**Figure 1:**
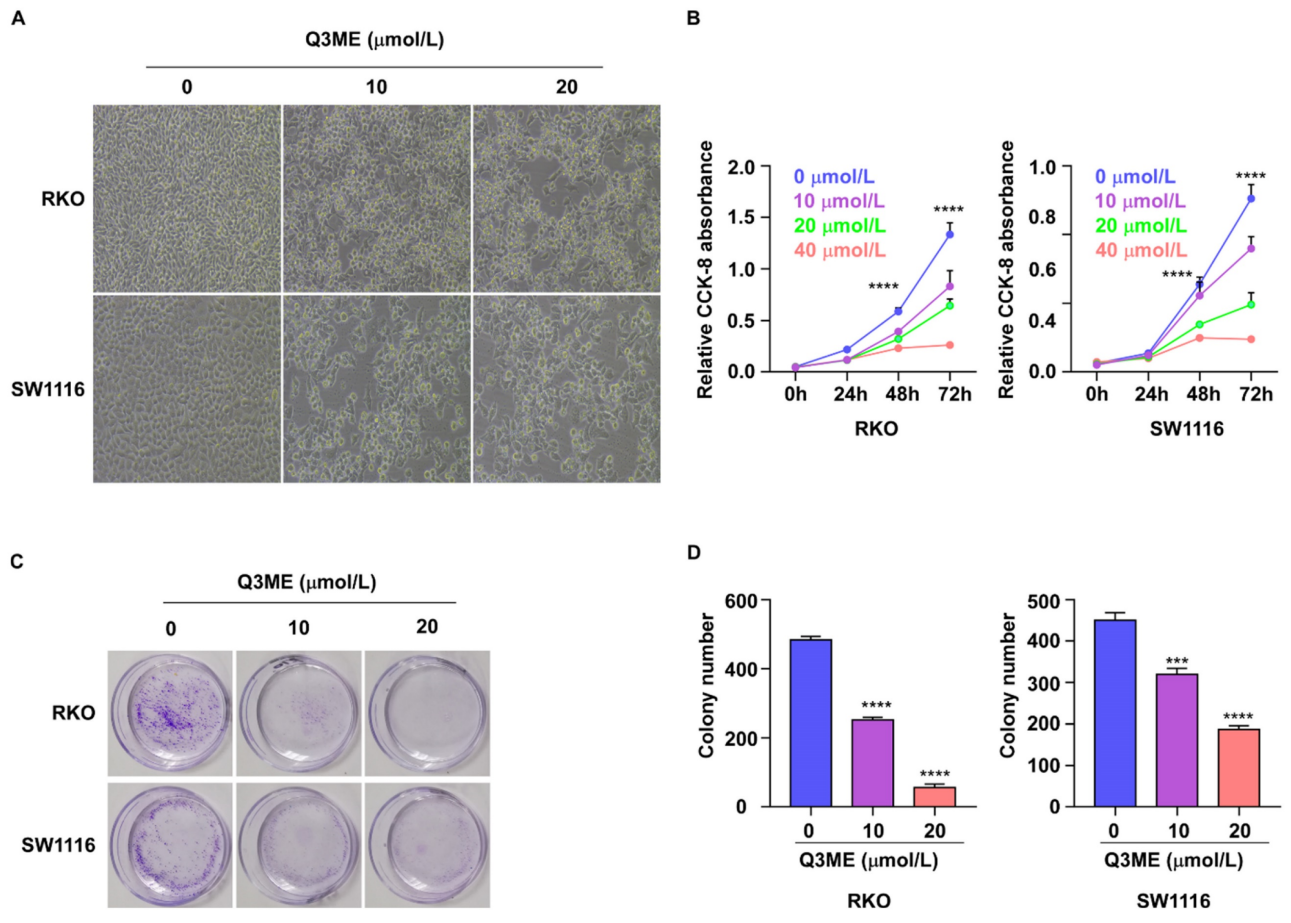
** Quercetin-3-methyl ether inhibits the viability of colorectal cancer cells. (A)** RKO and SW1116 cells were treated with 0-20 μM Quercetin-3-methyl ether for 72 h and cell morphology was observed under a microscope. **(B)** RKO and SW1116 cells were treated with increasing concentrations of Quercetin-3-methyl ether for 0-72 h and the cell viability was detected by CCK-8 assay. **(C)** RKO and SW1116 cells were treated with different concentrations of Quercetin-3-methyl ether and cell colonies were stained with crystal violet staining solution. **(D)** Quantitative analysis of the crystal violet-stained cells. ***P < 0.001, ****P < 0.0001.

Immortal viability is one of the malignant features of cancer cells. To this end, we examined the effect of Q3ME on the viability of colorectal cancer cells using the CCK-8 assay and colony formation assays. Colorectal cancer cells were treated with different concentrations of Q3ME at different times. The results showed that decreased cell viability was observed under the microscope after Q3ME treatment (Figure 1A). The results of CCK-8 assay showed that the viability of colorectal cancer cells was slightly inhibited after 24 hours of treatment with Q3ME (Figure 1B), and with the prolongation of drug treatment time, cell viability was significantly inhibited with the concentration of 40 μM (Figure 1B). Considering that the cell viability was significantly inhibited when treated with Q3ME concentration of 40 μM for 72 hours, 0 μM, 10 μM and 20 μM were selected for subsequent experiments. The colony formation assay was consistent with the CCK-8 results that Q3ME significantly inhibited the viability of colorectal cancer cells (Figure 1C and D). Thus, Q3ME inhibited the viability of colorectal cancer cells in a dose- and time-dependent manner.

### 3.2. Quercetin-3-methyl ether promotes apoptosis of colorectal cancer cells

**Figure 2:**
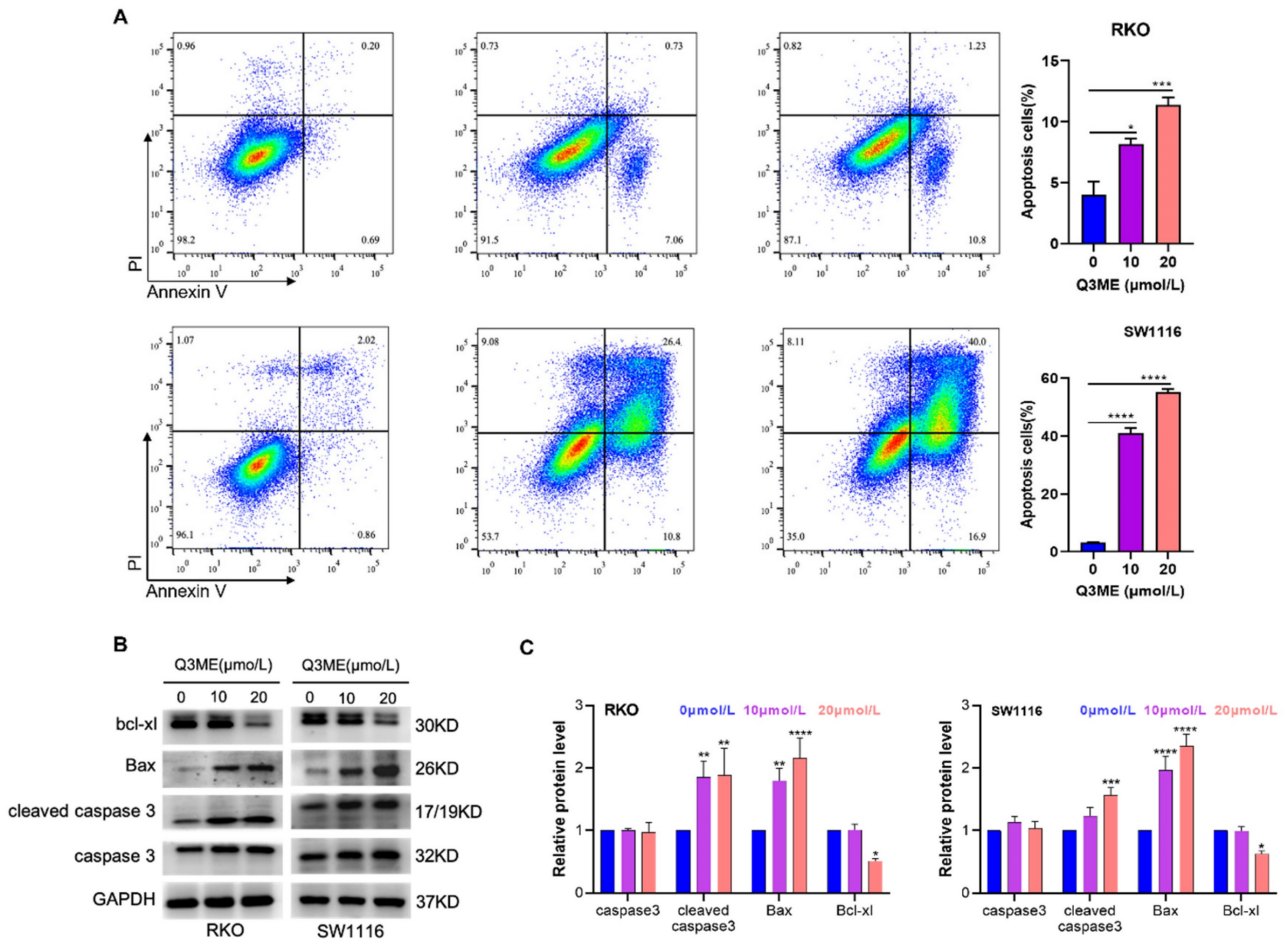
**Quercetin-3-methyl ether promotes apoptosis of colorectal cancer cells. (A)** RKO and SW1116 cells treated with Quercetin-3-methyl ether for 48 h were detected by flow cytometry using Annexin V FITC/PI staining. **(B)** Western blotting was used to detect the levels of Bad, Bcl-xl and Caspase-3 in RKO and SW1116 cells treated with Quercetin-3-methyl ether for 48 h. **(C)** Representative semiquantification of protein band densities normalized to GAPDH. *P < 0.05, **P < 0.01, ***P < 0.001, ****P < 0.0001.

Our previous study showed that Q3ME significantly inhibited the viability and clonal formation of lapatinib-sensitive and drug-resistant cell lines in breast cancer cells, induced G2/M phase arrest and promoted apoptosis 37. It has been demonstrated that Q3ME inhibits the viability of colorectal cancer cells in a dose- and time-dependent manner. In order to determine whether Q3ME can induce apoptosis of colorectal cancer cells, the number of apoptotic cells was detected by flow cytometry after treatment with different concentrations of Q3ME. The results showed that apoptosis of colorectal cancer cells increased with the increase of Q3ME concentration (Figure 2A), especially when the concentration of Q3ME was 20 μM, the apoptosis rate reached 12.03% (RKO) and 56.9% (SW1116). The difference of 44.87% in the apoptosis rate of the two colorectal cancer cell lines may be due to the different degree of differentiation between the cell lines. RKO cells are poorly differentiated cell lines, while SW1116 cells are moderately differentiated cell lines. Therefore, it could be speculated that Q3ME might have a stronger apoptosolytic effect on well-differentiated and moderately differentiated colorectal cancer cell lines. Western Blot results echoed flow cytometry results, and Q3ME could promote the expression of Bax and Cleaved caspase3, and inhibit the expression of anti-apoptotic protein Bcl-xl (Figure 2B and C). These findings suggest that Q3ME inhibits CRC cell viability and promotes apoptosis.

### 3.3. Quercetin-3-methyl ether reduces the content of spermine and spermidine in colorectal cancer cells

**Figure 3:**
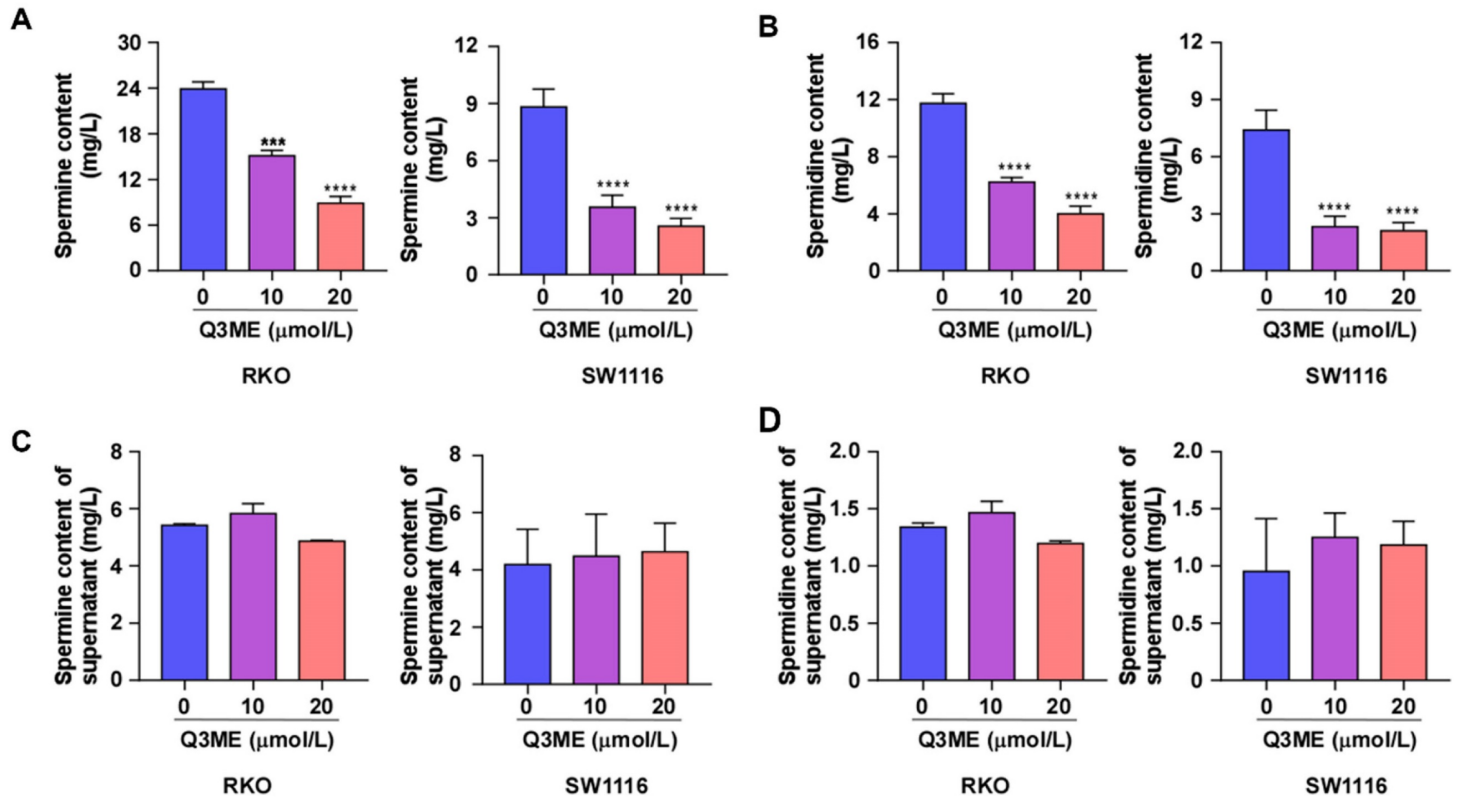
**Quercetin-3-methyl ether reduces the content of spermine and spermidine in colorectal cancer cells. (A)** and** (B)** After treating RKO and SW1116 cells with Quercetin-3-methyl ether for 48 hours, the contents of spermine and spermidine in the cells were determined by HPLC. **(C)** and** (D)** RKO and SW1116 cells were treated with Quercetin-3-methyl ether for 48 hours. The contents of spermine and spermidine in culture supernatant were determined by HPLC. ***P < 0.001, ****P < 0.0001.

Polyamines are necessary for normal cell growth, and their consumption can cause cell stagnation. In the early stages of tumor transformation and progression, multiple carcinogenic pathways lead to polyamine demand and metabolic dysregulation, suggesting that elevated polyamine levels are necessary for tumor progression 20, 39. Whether Q3ME affects the viability of colorectal cancer cells and promotes cell apoptosis through the polyamine metabolic pathway has not been reported. To this end, we treated colorectal cancer cells with different concentrations of Q3ME to detect the intracellular and extracellular polyamine content (spermine and spermidine) by High Performance Liquid Chromatography. The results showed that Q3ME decreased the levels of spermine and spermidine in colorectal cancer cells (Figure 3A and B), while the concentrations of spermine and spermidine in cell supernatant did not change (Figure 3C and D). These results suggest that Q3ME may promote the apoptosis of colorectal cancer cells by affecting polyamine metabolism.

### 3.4. Quercetin-3-methyl ether inhibits the expression of SMOX, a key enzyme of polyamine metabolism

**Figure 4:**
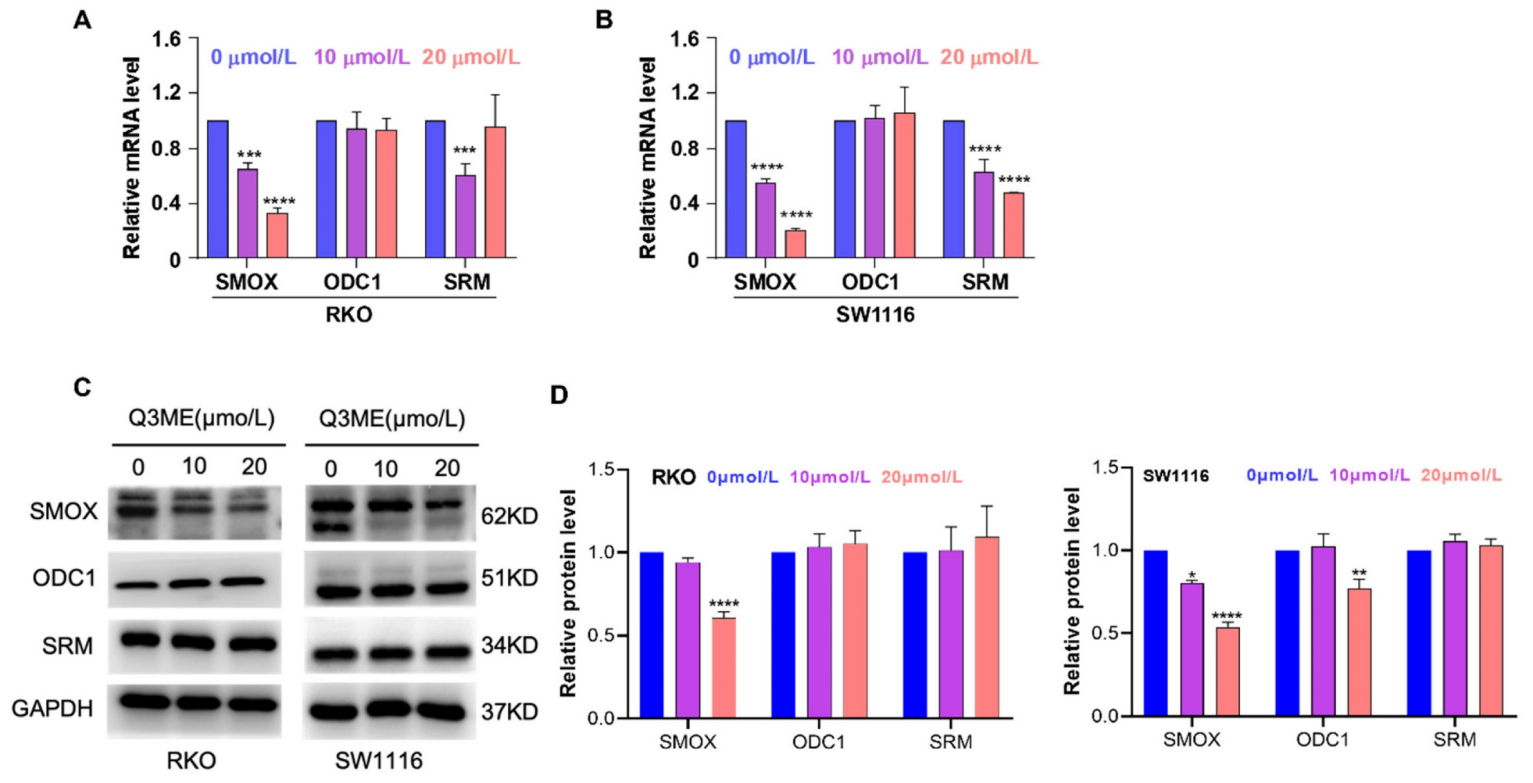
** Quercetin-3-methyl ether inhibits the expression of SMOX, a key enzyme of polyamine metabolism. (A)** The mRNA expression of *SMOX*, *ODC1* and *SRM* in RKO cells treated with Quercetin-3-methyl ether for 48 h was detected by RT-PCR. **(B)** The mRNA expression of *SMOX*, *ODC1* and *SRM* in SW1116 cells treated with Quercetin-3-methyl ether for 48 h was detected by RT-PCR. **(C)** Western blot was used to detect the expression levels of SMOX, ODC1 and SRM in RKO and SW1116 cells treated with Quercetin-3-methyl ether for 48 h. **(D)** Representative semiquantification of protein band densities normalized to GAPDH. *P < 0.05, **P < 0.01, ***P < 0.001, ****P < 0.0001.

SMOX is an FAD (Flavin adenine dinucleotide)-dependent enzyme that catalyzes SPM into the aldehyde 3-aminopropanal, H_2_O_2,_ and SPD, and reactive oxygen species are also produced in this process 40. Studies have shown that reactive oxygen species induced by oxidative stress can lead to apoptosis of epithelial cells and increase DNA damage, thereby increasing the risk of tumorigenesis 41. In addition, SMOX is upregulated in a variety of tumors, infections and inflammations, such as gastric cancer, liver cancer, etc. 42, 43. These suggest that SMOX is a plausible drug target for cancer therapy. To explore the potential role of SMOX in the development of colorectal cancer, we treated colorectal cancer cells with different concentrations of Q3ME to detect the mRNA and protein expression of related genes in the polyamine metabolic pathway. The results showed that Q3ME inhibited the mRNA and protein levels of SMOX, but had no effect on ODC1 and SRM (Figure 4A-D).

## 4. Discussion

Colorectal cancer is a malignant tumor caused by changes in genetic, epigenetic and environmental factors 44. The standard conventional treatments for CRC are surgery, chemotherapy, and radiation. Depending on disease localization and progression, these treatments can be used in combination 45-48. In recent decades, many forms of complementary and alternative medicine have been used worldwide to fight cancer. Traditional Chinese medicine (TCM) is a huge resource for the development of anti-cancer drugs. The treatment of tumors with traditional Chinese medicine has the advantages of multiple targets, multiple pathways and integrity. Traditional Chinese medicine and its active molecules can inhibit the viability of tumor cells by regulating several important signal transduction pathways, including apoptosis, autophagy and polyamine metabolism 49-51. Quercetin-3-methyl ether is a flavonoid widely present in traditional Chinese medicinal materials such as Bupleurum and Mulberry leaves, and is widely present in nature and daily diet 28. Quercetin-3-methyl ether has been reported to inhibit the growth of a variety of malignancies, however, little is known about the functional effects of this compound on CRC cells and its antitumor mechanism. In this study, a variety of methods were applied to examine the effect of Quercetin-3-methyl ether on the viability and apoptosis of colorectal cancer cells. It is showed that Q3ME inhibited colorectal cancer cells viability in a dose- and time-dependent manner, and that Q3ME promoted the apoptosis of colorectal cancer cells. Metabolic reprogramming represents a fundamental feature of most cancer cells.

**Figure 5:**
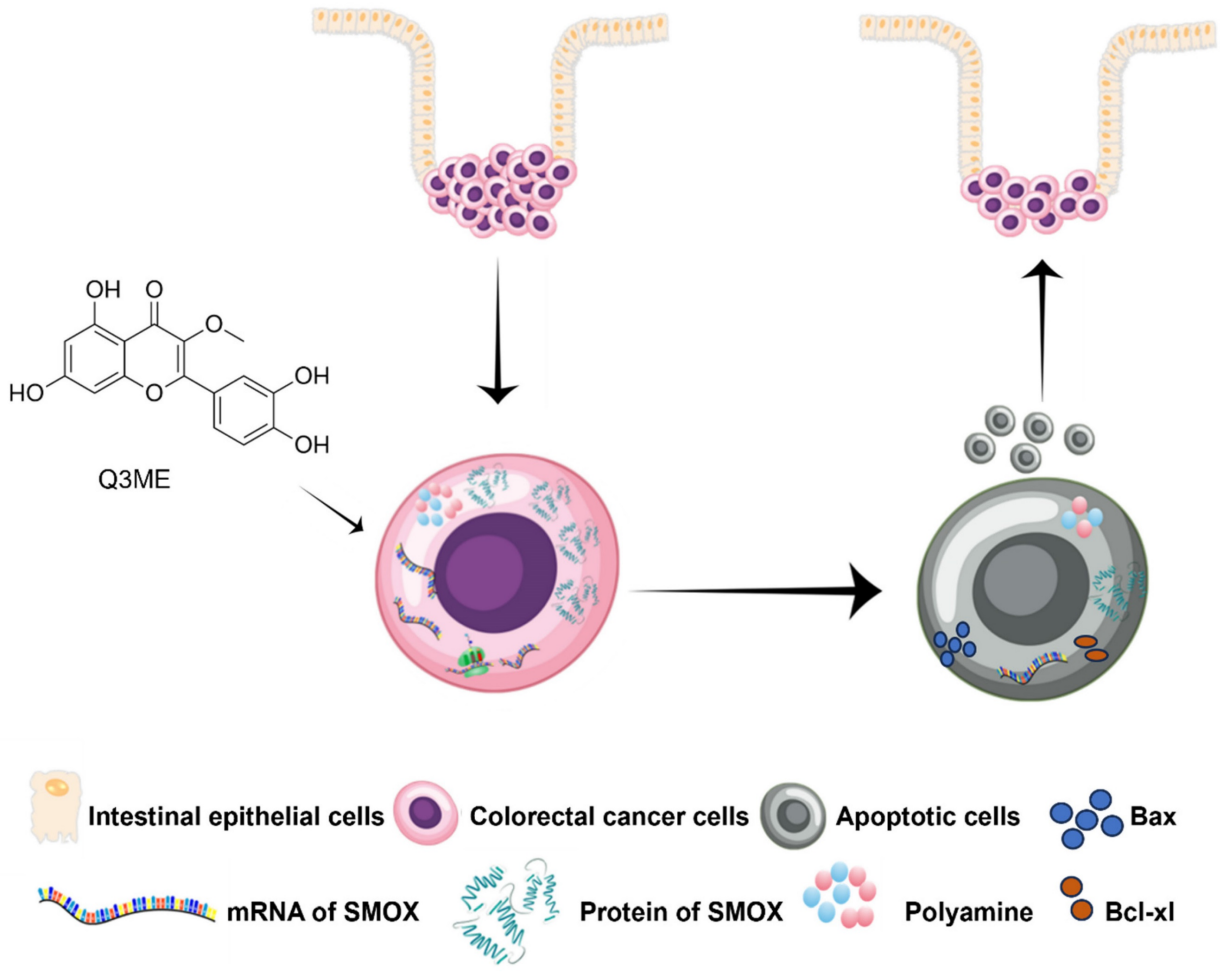
** Simplified diagram of the proposed anticancer mechanism of Quercetin-3-methyl ether in Colorectal cancer based on the results of the present study.** Quercetin-3-methyl ether may inhibit the level of polyamines in colorectal cancer cells and promote colorectal cancer cell apoptosis by regulating the expression of SMOX.

In fact, most of the core metabolic pathways are utilized by cancer cells to maintain their high cell division rates 52. In addition to cell viability, it is becoming increasingly clear that cell metabolism is closely related to the fate and phenotype of cancer cells, which control the epigenetic inheritance of tumor cells and how these cells interact with their surrounding microenvironment. Importantly, these metabolic interactions are key to regulating tumor progression and shaping its response to chemotherapy 8, 53. Cancer metabolism, especially polyamine metabolism, is a major factor in tumorigenesis, growth, metastasis and diffusion in CRC 54. Polyamines are necessary for normal cell growth, and their depletion can cause cell stagnation. In the early stages of tumor transformation and progression, multiple carcinogenic pathways lead to polyamine demand and metabolic dysregulation 39. At present, accumulating evidence suggests that polyamine metabolism is a target of anticancer therapy 55, 56. It is worth noting that SMOX ectopic expression in neuroblastoma cells increases oxidative DNA damage thus inducing apoptosis 57. A recent study has shown that SMOX is significantly upregulated in CRC cell lines and clinical tissues. The overexpression of SMOX in tumor tissues has been identified as an independent prognostic factor, leading to a poorer overall survival rate. Furthermore, the suppression of SMOX has been found to inhibit the proliferation of CRC cells and their ability to form colonies, revealing its role in promoting carcinogenic functions 58. Therefore, polyamine metabolism of colorectal cancer cells was evaluated to further explore the potential molecular mechanism under the tumor inhibitory effect of Q3ME. In our study, we first investigated whether or not culturing with Q3ME impacts viability and colony formation of RKO and SW1116 cells. Our results showed that Q3ME could suppress viability and colony formation of RKO and SW1116 cells. Q3ME decreased the levels of polyamines (spermine and spermidine) in colorectal cancer cells, and reduced the protein expression of SMOX, an enzyme responsible for converting spermine to spermidine. This suggests that Q3ME may induce apoptosis in colorectal cancer cells by decreasing intracellular spermidine level through the inhibition of SMOX (Figure 5). Additionally, Q3ME upregulated the levels of pro-apoptosis protein bax and caspase 3 while downregulating the expression level of anti-apoptosis protein bcl-xl in colorectal cancer cells. These results suggest that Q3ME may promote the apoptosis of colorectal cancer cells by inhibiting the expression of SMOX.

These findings provide new insights into a better understanding of the pharmacological activity of Quercetin-3-methyl ether as a natural product and how it reprograms cellular polyamine metabolism.

## References

[1] Sung H, Ferlay J, Siegel RL, Laversanne M, Soerjomataram I, Jemal A (2021). Global Cancer Statistics 2020: GLOBOCAN Estimates of Incidence and Mortality Worldwide for 36 Cancers in 185 Countries. CA Cancer J Clin.

[2] Xi Y, Xu P (2021). Global colorectal cancer burden in 2020 and projections to 2040. Transl Oncol.

[3] Keum N, Giovannucci E (2019). Global burden of colorectal cancer: emerging trends, risk factors and prevention strategies. Nat Rev Gastroenterol Hepatol.

[4] Chatila WK, Kim JK, Walch H, Marco MR, Chen CT, Wu F (2022). Genomic and transcriptomic determinants of response to neoadjuvant therapy in rectal cancer. Nat Med.

[5] Wang R, Li J, Zhou X, Mao Y, Wang W, Gao S (2022). Single-cell genomic and transcriptomic landscapes of primary and metastatic colorectal cancer tumors. Genome Med.

[6] Brenner H, Kloor M, Pox CP (2014). Colorectal cancer. Lancet.

[7] Park JH, Richards CH, McMillan DC, Horgan PG, Roxburgh CSD (2014). The relationship between tumour stroma percentage, the tumour microenvironment and survival in patients with primary operable colorectal cancer. Ann Oncol.

[8] Vander Heiden MG, DeBerardinis RJ (2017). Understanding the Intersections between Metabolism and Cancer Biology. Cell.

[9] Pavlova NN, Thompson CB (2016). The Emerging Hallmarks of Cancer Metabolism. Cell Metab.

[10] Casero RA Jr, Marton LJ (2007). Targeting polyamine metabolism and function in cancer and other hyperproliferative diseases. Nat Rev Drug Discov.

[11] Miller-Fleming L, Olin-Sandoval V, Campbell K, Ralser M (2015). Remaining Mysteries of Molecular Biology: The Role of Polyamines in the Cell. J Mol Biol.

[12] Pegg AE (2016). Functions of Polyamines in Mammals. J Biol Chem.

[13] Lee CY, Su GC, Huang WY, Ko MY, Yeh HY, Chang GD (2019). Promotion of homology-directed DNA repair by polyamines. Nat Commun.

[14] Li H, Wu BK, Kanchwala M, Cai J, Wang L, Xing C (2022). YAP/TAZ drives cell proliferation and tumour growth via a polyamine-eIF5A hypusination-LSD1 axis. Nat Cell Biol.

[15] Bae DH, Lane DJR, Jansson PJ, Richardson DR (2018). The old and new biochemistry of polyamines. Biochim Biophys Acta Gen Subj.

[16] Babbar N, Murray-Stewart T, Casero RA Jr (2007). Inflammation and polyamine catabolism: the good, the bad and the ugly. Biochem Soc Trans.

[17] Park MH, Igarashi K (2013). Polyamines and their metabolites as diagnostic markers of human diseases. Biomol Ther (Seoul).

[18] Igarashi K, Ueda S, Yoshida K, Kashiwagi K (2006). Polyamines in renal failure. Amino Acids.

[19] Kramer DL, Diegelman P, Jell J, Vujcic S, Merali S, Porter CW (2008). Polyamine acetylation modulates polyamine metabolic flux, a prelude to broader metabolic consequences. J Biol Chem.

[20] Casero RA Jr, Murray Stewart T, Pegg AE (2018). Polyamine metabolism and cancer: treatments, challenges and opportunities. Nat Rev Cancer.

[21] Levin VA, Ictech SE, Hess KR (2018). Clinical importance of eflornithine (alpha-difluoromethylornithine) for the treatment of malignant gliomas. CNS Oncol.

[22] Raj KP, Zell JA, Rock CL, McLaren CE, Zoumas-Morse C, Gerner EW (2013). Role of dietary polyamines in a phase III clinical trial of difluoromethylornithine (DFMO) and sulindac for prevention of sporadic colorectal adenomas. Br J Cancer.

[23] Murray-Stewart T, Ferrari E, Xie Y, Yu F, Marton LJ, Oupicky D (2017). Biochemical evaluation of the anticancer potential of the polyamine-based nanocarrier Nano11047. PLoS One.

[24] Xie Y, Murray-Stewart T, Wang Y, Yu F, Li J, Marton LJ (2017). Self-immolative nanoparticles for simultaneous delivery of microRNA and targeting of polyamine metabolism in combination cancer therapy. J Control Release.

[25] Hayes CS, Shicora AC, Keough MP, Snook AE, Burns MR, Gilmour SK (2014). Polyamine-blocking therapy reverses immunosuppression in the tumor microenvironment. Cancer Immunol Res.

[26] Cui Y, Shu XO, Gao Y, Wen W, Ruan ZX, Jin F (2004). Use of complementary and alternative medicine by chinese women with breast cancer. Breast Cancer Res Treat.

[27] Chen Z, Gu K, Zheng Y, Zheng W, Lu W, Shu XO (2008). The use of complementary and alternative medicine among Chinese women with breast cancer. J Altern Complement Med.

[28] Martino R, Barreiro Arcos ML, Peralta I, Marrassini C, Saint Martin EM, Cogoi L (2021). Antiproliferative activity of aqueous and polyphenol-rich extracts of Larrea divaricata Cav. on a melanoma cell line. Nat Prod Res.

[29] Ustun O, Ozcelik B, Akyon Y, Abbasoglu U, Yesilada E (2006). Flavonoids with anti-Helicobacter pylori activity from Cistus laurifolius leaves. J Ethnopharmacol.

[30] Smolarz HD, Surdacka A, Rolinski J (2003). Influence of ethyl acetate extract and quercetin-3-methyl ether from Polygonum amphibium on activation lymphocytes from peripheral blood of healthy donor in vitro. Phytother Res.

[31] Iheagwam FN, Ogunlana OO, Ogunlana OE, Isewon I, Oyelade J (2019). Potential Anti-Cancer Flavonoids Isolated From Caesalpinia bonduc Young Twigs and Leaves: Molecular Docking and In Silico Studies. Bioinform Biol Insights.

[32] Yang GG, Zhou DJ, Pan ZY, Yang J, Zhang DY, Cao Q (2019). Multifunctional low-temperature photothermal nanodrug with in vivo clearance, ROS-Scavenging and anti-inflammatory abilities. Biomaterials.

[33] Yuan Y, Zheng S, Zeng L, Deng Z, Zhang B, Li H (2019). The Phenolic Compounds, Metabolites, and Antioxidant Activity of Propolis Extracted by Ultrasound-Assisted Method. J Food Sci.

[34] Sun Y, Li C, Li Z, Shangguan A, Jiang J, Zeng W (2021). Quercetin as an antiviral agent inhibits the Pseudorabies virus in vitro and in vivo. Virus Res.

[35] Yan L, Vaghari-Tabari M, Malakoti F, Moein S, Qujeq D, Yousefi B (2022). Quercetin: an effective polyphenol in alleviating diabetes and diabetic complications. Crit Rev Food Sci Nutr.

[36] Wang Y, Li C, Wan Y, Qi M, Chen Q, Sun Y (2021). Quercetin-Loaded Ceria Nanocomposite Potentiate Dual-Directional Immunoregulation via Macrophage Polarization against Periodontal Inflammation. Small.

[37] Cao L, Yang Y, Ye Z, Lin B, Zeng J, Li C (2018). Quercetin3methyl ether suppresses human breast cancer stem cell formation by inhibiting the Notch1 and PI3K/Akt signaling pathways. Int J Mol Med.

[38] Martino R, Arcos ML, Alonso R, Sulsen V, Cremaschi G, Anesini C (2016). Polyphenol-Rich Fraction from Larrea divaricata and its Main Flavonoid Quercetin-3-Methyl Ether Induce Apoptosis in Lymphoma Cells Through Nitrosative Stress. Phytother Res.

[39] Murray-Stewart TR, Woster PM, Casero Jr RA (2016). Targeting polyamine metabolism for cancer therapy and prevention. Biochemical journal.

[40] Wang Y, Devereux W, Woster PM, Stewart TM, Hacker A, Casero RA (2001). Cloning and characterization of a human polyamine oxidase that is inducible by polyamine analogue exposure. Cancer research.

[41] Chaturvedi R, Asim M, Romero-Gallo J, Barry DP, Hoge S, De Sablet T (2011). Spermine oxidase mediates the gastric cancer risk associated with Helicobacter pylori CagA. Gastroenterology.

[42] Chaturvedi R, de Sablet T, Asim M, Piazuelo MB, Barry DP, Verriere TG (2015). Increased Helicobacter pylori-associated gastric cancer risk in the Andean region of Colombia is mediated by spermine oxidase. Oncogene.

[43] Hu T, Sun D, Zhang J, Xue R, Janssen HL, Tang W (2018). Spermine oxidase is upregulated and promotes tumor growth in hepatocellular carcinoma. Hepatology Research.

[44] Bray F, Ferlay J, Soerjomataram I, Siegel RL, Torre LA, Jemal A (2018). Global cancer statistics 2018: GLOBOCAN estimates of incidence and mortality worldwide for 36 cancers in 185 countries. CA: a cancer journal for clinicians.

[45] Schmoll H, Van Cutsem E, Stein A, Valentini V, Glimelius B, Haustermans K (2012). ESMO Consensus Guidelines for management of patients with colon and rectal cancer. a personalized approach to clinical decision making. Annals of oncology.

[46] Van Cutsem E, Cervantes A, Nordlinger B, Arnold D (2014). Metastatic colorectal cancer: ESMO Clinical Practice Guidelines for diagnosis, treatment and follow-up. Annals of oncology.

[47] Van Cutsem E, Cervantes A, Adam R, Sobrero A, Van Krieken J, Aderka D (2016). ESMO consensus guidelines for the management of patients with metastatic colorectal cancer. Annals of Oncology.

[48] Yoshino T, Arnold D, Taniguchi H, Pentheroudakis G, Yamazaki K, Xu R-H (2018). Pan-Asian adapted ESMO consensus guidelines for the management of patients with metastatic colorectal cancer: a JSMO-ESMO initiative endorsed by CSCO, KACO, MOS, SSO and TOS. Annals of Oncology.

[49] Wang S-F, Wu M-Y, Cai C-Z, Li M, Lu J-H (2016). Autophagy modulators from traditional Chinese medicine: Mechanisms and therapeutic potentials for cancer and neurodegenerative diseases. Journal of ethnopharmacology.

[50] Hernandez-Valencia J, Garcia-Villa E, Arenas-Hernandez A, Garcia-Mena J, Diaz-Chavez J, Gariglio P (2018). Induction of p53 phosphorylation at serine 20 by resveratrol is required to activate p53 target genes, restoring apoptosis in MCF-7 cells resistant to cisplatin. Nutrients.

[51] Zhang Z, Li H, Li W, Feng Y, Hu Z, Zhou S (2020). Evidence for polyamine, biogenic amine, and amino acid adduction resulting from metabolic activation of diosbulbin B. Chemical Research in Toxicology.

[52] Pavlova NN, Thompson CB (2016). The emerging hallmarks of cancer metabolism. Cell metabolism.

[53] Rinaldi G, Rossi M, Fendt SM (2018). Metabolic interactions in cancer: cellular metabolism at the interface between the microenvironment, the cancer cell phenotype and the epigenetic landscape. Wiley Interdisciplinary Reviews: Systems Biology and Medicine.

[54] Muir A, Danai LV, Vander Heiden MG (2018). Microenvironmental regulation of cancer cell metabolism: implications for experimental design and translational studies. Disease models & mechanisms.

[55] Arruabarrena-Aristorena A, Zabala-Letona A, Carracedo A (2018). Oil for the cancer engine: The cross-talk between oncogenic signaling and polyamine metabolism. Science advances.

[56] Casero RA (2018). Targeting the aryl hydrocarbon receptor/polyamine biosynthesis axis of evil for cancer therapy. The Journal of Clinical Investigation.

[57] Fratini E, Cervelli M, Mariottini P, Kanamori Y, Amendola R, Agostinelli E (2019). Link between spermine oxidase and apoptosis antagonizing transcription factor: A new pathway in neuroblastoma. International journal of oncology.

[58] Kim S, Kim D, Roh S, Hong I, Kim H, Ahn TS (2022). Expression of Spermine Oxidase Is Associated with Colorectal Carcinogenesis and Prognosis of Patients. Biomedicines.

